# The Cell Surface Markers Expression in Postmenopausal Women and Relation to Obesity and Bone Status

**DOI:** 10.3390/ijerph14070751

**Published:** 2017-07-11

**Authors:** Mira Horváthová, Silvia Ilavská, Kornélia Štefíková, Michaela Szabová, Zora Krivošíková, Eva Jahnová, Jana Tulinská, Viera Spustová, Martin Gajdoš

**Affiliations:** 1 Department of Immunology and Immunotoxicology, Faculty of Medicine, Slovak Medical University, 833 03 Bratislava, Slovakia; silvia.ilavska@szu.sk (S.I.); michaela.szabova@szu.sk (M.S.); eva.jahnova@szu.sk (E.J.); jana.tulinska@szu.sk (J.T.); 2 Department of Clinical and Experimental Pharmacology, Faculty of Medicine, Slovak Medical University, 83303 Bratislava, Slovakia; kornelia.stefikova@szu.sk (K.Š.); zora.krivosikova@szu.sk (Z.K.); viera.spustova@szu.sk (V.S.); martin.gajdos@szu.sk (M.G.)

**Keywords:** cell surface molecules, postmenopausal women, obesity, bone status, inflammation

## Abstract

The age-related changes and hormonal deprivation in postmenopausal women are associated with the immune response alteration. The excessive fat accumulation, local and systemic inflammation may lead to dysregulation in immune function and relevant health problems, including obesity and osteoporosis. We analyzed the expression of cell surface markers in the venous blood specimens, stained with fluorophores-conjugated monoclonal antibodies and analysed by multicolour flow cytometry. The significant changes of cytotoxic, naive, and memory T-lymphocytes, plasmacytoid dendritic cells (DCs) were in postmenopausal women versus fertile women. Body mass index (BMI) affected markedly the cell surface expression of CD265/RANK. Osteoporosis is linked to reduced percentage of plasmacytoid DCs, and elevated natural Treg cells (*p* < 0.05). The confounding factors such as women age, BMI, bone mineral density (BMD), waist size and tissue fat affect the expression of RANK on myeloid DCs and CD40L on T-lymphocytes that might be the immunophenotypic modulators after menopause.

## 1. Introduction

The presence of immune response dysregulation is related to many health alterations in postmenopausal condition. Menopause changes in the hormone levels are associated with an elevated body fat, high blood pressure, insulin resistance and dyslipidemia. With aging, a general decline in immune function is observed leading to immune senescence. Several of these changes affecting postmenopausal women are gender specific. Age and female sex increase the risk of obesity, metabolic syndrome and osteoporosis developing. The hallmarks for immune senescence include changes in T cell ratio, memory and naive T-lymphocytes, effector T- and B-lymphocytes. Postmenopausal women show an inflammatory immune microenvironment, reduced ability to respond to pathogens or stimuli, decreased cytotoxic activity of natural killer (NK) cells, higher chronic pro-inflammatory cytokines production. The infections are more common in this group as a result of attenuated immune response and higher susceptibility to pathogenic invasion [1,2,3].

During the last decades, obesity has become important global health problem with an increasing prevalence and a high impact on both mortality and morbidity worldwide. Obesity probably interfering with bone health, the existence of a cross talk between fat and the skeleton suggests a homeostatic feedback system in which adipokines and bone-derived molecules form part of an active bone [4]. New evidences of the relationship between immune system and bone have been accumulated both in animal models and in humans affected by bone disease. Osteoporosis is characterized by low bone mass and microarchitectural deterioration of bone tissue with a subsequent increase in bone fragility and susceptibility to fractures. The combined effects of the changed production of hormones (estrogen, follicle-stimulating hormone (FSH)) occurring in menopause cause a marked stimulation of bone resorption and a rapid bone loss which is central for the onset of postmenopausal osteoporosis. There is a role of T- and B-lymphocytes in the human postmenopausal bone loss [5]. The osteoclastogenic RANKL (receptor activator of nuclear factor κ-B ligand, CD254) production by T-lymphocytes may contribute to bone loss in humans. RANKL molecule and its receptor RANK (receptor activator of nuclear factor κ-B, CD265) are key regulators of bone remodeling, and they are essential for the development and activation of osteoclasts. RANKL/RANK interactions also regulate T cells–dendritic cells (DCs) communications. The RANKL/RANK pathway is involved in inflammatory process, and is critical in bone destruction [6,7]. A number of studies have established that RANKL control bone regeneration and remodelling, this effect is mediated through the induction of regulatory T (Treg) cells by osteoclasts [8,9,10].

DCs affect onset and progression of chronic inflammatory diseases. Acting as sentinel immune cells, DCs initiate, coordinate, and regulate a wide variety of immune responses. The postmenopausal changes have an impact on dendritic function in women. Immune cells, DCs and macrophages, participate in T and B cell differentiation [11,12]. NK cells and natural killer T (NKT) cells play an important role in regulating innate and adaptive immune response, their cytotoxicity being effective to kill infected cells and even tumors. Age-related alterations of NK and NKT cells in their numbers and activities have been reported. The excessive fat accumulation leads to substantial changes in the amount and function of immune cells, some of them increase the number and activity (e.g., macrophages, T- and B-lymphocytes), while others simultaneously reduced, including several subsets of T- lymphocytes (e.g., Treg, NKT cells). This imbalance lies at the very core of the development of obesity-related local and systemic inflammation. The number of circulating Treg cells are inversely correlated with body mass index (BMI) and these cells are significantly reduced in obese subjects [13,14,15].

In a recent study we compared the expression of cell surface markers in whole blood from fertile and postmenopausal women. The objective of this study was an examination of T- and B-lymphocytes, NK cells, NKT cells, activated T-lymphocytes, memory and naive T-lymphocytes, natural Treg cells, plasmacytoid and myeloid DCs. We also evaluated the expression of RANK receptor on DCs and RANKL on T-lymphocytes. Additionally, the potential confounding effects such as women age, BMI, bone mineral density (BMD), waist size and tissue fat were taken into account in multivariate linear regression models.

## 2. Materials and Methods

### 2.1. Study Population

The blood specimens were obtained from Slovak women (*n* = 203), of this fertile (*n* = 96; age range 26–52 years) and postmenopausal (*n* = 107; age range 48–79 years). Each group was distributed into two subgroups according to their BMI value: normal body weight (control group; BMI = 20.0–29.9 kg/m^2^) and obese subjects (BMI ≥ 30.0 kg/m^2^). The reproductive status (fertile or postmenopausal) in adult women was determined anamnestically. Postmenopausal women were at least five years since the beginning of menopause, the case of natural menopause (*n* = 82, duration of menopause 5–27 years) or women after artificial menopause, i.e., simple hysterectomy (*n* = 0), hysterectomy with bilateral (*n* = 12, duration of menopause 5–35 years) or unilateral ovariectomy (*n* = 1, 6 years in menopause). Some of women were treated for hypertension (*n* = 49), most of them postmenopausal (*n* = 43). None of women were treated for osteopenia or osteoporosis in the past and they had no history of femur or vertebral fractures. Subjects were recruited via health practitioners in accordance with ethical committee requirements and completed a detailed nutritional and lifestyle questionnaire and psychological tests. Criteria for inclusion were no acute physical illness or unstable physical condition. Criteria for exclusion were pregnancy, daily smoking for over a one year, nephropathy with glomerular filtration rate (GFR < 0.75 mL/s), endocrinopathy, diabetes mellitus, active hepatitis and liver cirrhosis, cancers, anemia, severe cardiovascular disease, malabsorption diseases or conditions after gastrectomy or any part of the intestine, alcohol abuse, drug addiction, treatment with glucocorticoids, hormone therapy or hormone replacement therapy, use of calcium, vitamin D (at a dose more than 400 IU), drugs affecting obesity, the presence of metal implants and pacemaker in the body. All subjects underwent a full physical examination including measurements of height, weight, waist circumference. Body weight was measured with a conventional balance and height was measured using a wall-mounted Harpenden stadiometer (Holtain LTD., Crymych, UK). As an indicator of central adiposity, waist circumference was measured to the nearest 1 mm with a nonextensible measuring tape at the level of the umbilicus. Obesity was estimated by calculating the BMI (kg/m^2^) and the whole body composition was measured using a total-body scanner (Lunar Prodigy Advance with Encore 2011 software version 13.60 GE Medical Systems, Madison, WI, USA). In obese women half-scan measurement and analysis was performed, if the subject’s body was not contained within the scan space. A bone densitometry measurement was done at all participants at the first time, without knowledge of bone density in the past. BMD of the lumbar spine of area L1–L4, total femur and femoral neck were measured, using dual energy X-ray absorptiometry (DXA). The results were evaluated according to the WHO expert criteria. Osteopenia was defined by BMD −1 SD to −2.5 SD mean values of young healthy probands (T-score) and osteoporosis was defined by BMD < −2.5 SD, for postmenopausal women. Low BMD of fertile women was estimated by BMD < −2.0 SD mean values of age matched subjects (Z-score). All participants provided informed consent, approved by Ethical Committee (ECSMU 06102011).

### 2.2. Detection of Cell Surface Markers

The venous blood specimens were collected into EDTA vacutainer tubes. Each cell surface receptor of interest was analyzed by multi-color immunophenotyping using monoclonal antibodies CD4-FITC (fluorescein isothiocyanate, eBioscience, San Diego, CA, USA), CD3-FITC (Beckman, Brea, CA, USA), CD127-PE (phycoerythrin, Beckman), CD45RA-PE (Beckman), CD56-PE (Beckman), CD40L-PE (Beckman), CD45RO-ECD (electron coupled dye, Beckman), HLADR-ECD (Beckman), CD25-PC5 (phycoerythrin-cyanin 5, Beckman), CD8-PC5 (Beckman), CD28-PC5 (Beckman), CD19-PC7 (phycoerythrin-cyanin 7, Beckman), CD265-PE (Acris Antibodies, Herford, Germany), CD254-PE (BioLegend, San Diego, CA, USA), CD123-FITC (Exbio, Praha, Czech Republic), CD11c-APC (allophycocyanin, Exbio). Whole blood specimens were treated with OptiLyse-C Lysing solution (Beckman Coulter, Marseille, France) and washed with phosphate buffered saline (PBS, pH 7.4). Blood samples were stained according to manufacturer’s instructions and cell surface receptor analysis was performed using a Cytomics FC500 flow cytometer and CXP software (Beckman). Data were generated via gating through the lymphocyte and/or monocyte regions. NKT cells were defined as CD3+/CD56+ cells. An acquisition gate for DCs included all mononuclear cells. DCs were defined as the cells negative for anti-CD19 and positive for anti-HLA-DR. Monoclonal antibodies anti-CD11c and anti-CD123 were used for further identification of the myeloid and plasmacytoid DCs subsets. A 10,000 events per sample were acquired for analysis. Quality control procedure was performed using appropriate reference biological control (Immuno-Trolls Cells, Beckman). Fluorescent microspheres were used for cytometer alignment verification and fluorescence channel monitoring.

### 2.3. Statistical Analysis

Statistical analyses were performed using the SPSS statistical software package (SPSS Inc., Chicago, IL, USA). Non-parametric Mann-Whitney test was used for data evaluation, and comparison between subjects. Subject characteristics are described as mean ± SD. The primary hypothesis was that the cell surface molecules expression would differ between samples of fertile women compared to postmenopausal women. A multiple linear regression model was used for the evaluation of the potential confounders, such as women age, BMI, BMD, waist size and tissue fat. Each variable was entered in sequence and assessed by the retain criteria in the model. *p* < 0.05 represented statistical significance.

## 3. Results

**Figure 1:**
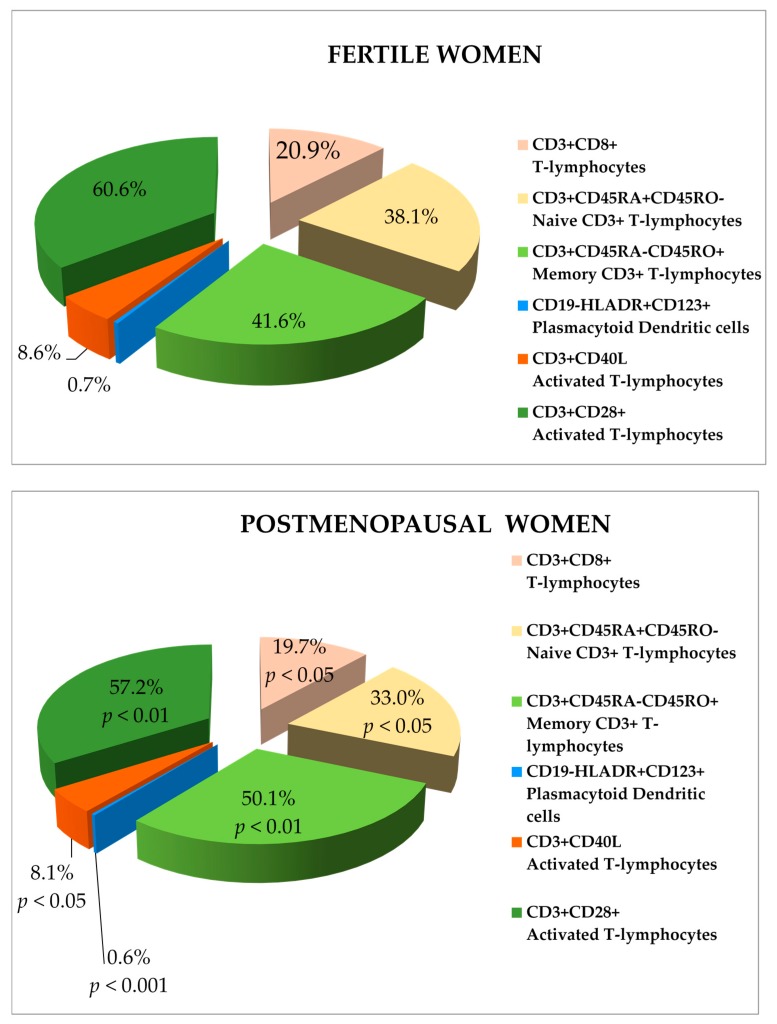
Comparison of the cell surface markers expression (%) in whole blood of fertile and postmenopausal women with statistical significant differences. CD = cluster of differentiation.

Table 1 reports the descriptive characteristics (mean, median, percentiles) of the total cell surface expression and cell populations from all women. Statistically significant differences between the fertile and postmenopausal women group have been demonstrated (Figure 1). 

The postmenopausal women presented significantly lower percentage of CD3+CD8+ T-lymphocytes, naive CD3+ T-lymphocytes and CD3+CD40L+ activated T-lymphocytes (*p* < 0.05), CD3+CD28+ activated T-lymphocytes (*p* < 0.01), plasmacytoid DCs (*p* < 0.001), and higher percentage of memory CD3+ T-lymphocytes (*p* < 0.01), compared to fertile women.

We have monitored the percentages of cell surface expression in four groups of women divided according the fertility and BMI parameters: (1) fertile obese group; (2) fertile control; (3) postmenopausal obese group; and (4) postmenopausal control. There were significant decrease of CD3+ T-lymphocytes, naive CD3+ T-lymphocytes, plasmacytoid DCs, CD265+ and CD265+CD11c+ monocyte population, CD3+CD40L+ and CD3+CD28+ activated T-lymphocytes in postmenopausal obese women compared to fertile obese group. CD19+ B-lymphocytes and naive CD3+ T-lymphocytes have diminished in postmenopausal control when compared to fertile control group (*p* < 0.05). The effect of BMI was observed between postmenopausal obese and non-obese women in the CD265+ and CD265+CD11c+ monocyte population (*p* < 0.05, *p* < 0.01, respectively), and plasmacytoid DCs (*p* < 0.05) (Table 2).

BMI, BMD, tissue fat, waist size, and age were also found to be associated with the cell surface receptor expression in the total study cohort. Significant positive correlation was between CD4+ T-lymphocytes and waist size, memory CD3+ T-lymphocytes and BMD.

**Figure 2:**
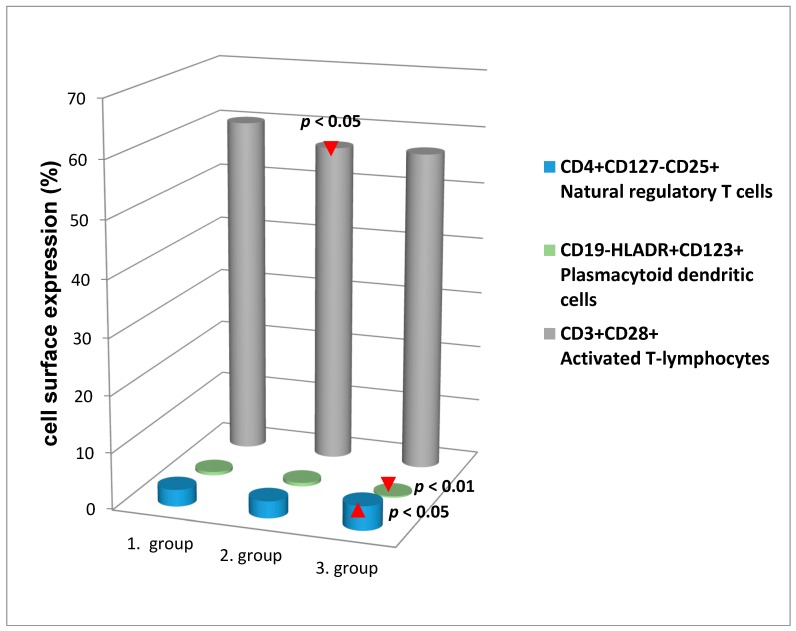
Significant changes of the cell surface expression between women with different bone mineral density. BMD = bone mineral density, 1. group = normal BMD (*n* = 126); 2. group = osteopenia (*n* = 62); 3. group = osteoporosis (*n* = 11).

We have found statistically significant negative associations in several cell populations, namely plasmacytoid and myeloid DCs, CD3+CD28+ activated T-lymphocytes, memory effector cells, naive CD3+ T-lymphocytes and RANK on plasmacytoid DCs (Table 3). The significant changes of cell surface expression in the women with different BMD are shown in Figure 2. The number of CD3+CD28+ activated T-lymphocytes and CD19-HLADR+CD123+ plasmacytoid DCs were meaningly lower in women with osteopenia (2. group) and osteoporosis (3. group), respectively, in comparison to 1. Group with normal BMD. The CD4+CD127-CD25+ natural Treg cell levels were increased in women affected by osteoporosis (3. group) (*p* < 0.05).

Results from multiple linear regression for immunophenotyping parameters are shown in Table 4. After adjustment of covariantes (BMD, BMI, tissue fat, waist size, women age), the level of RANK on myeloid DCs and CD3+CD40L+ T-lymphocytes remained significantly different between the fertile and postmenopausal women groups. The women age showed a significant effect on CD3+ and CD3+CD40L+ T-lymphocytes, RANK on myeloid DCs. Samples revealed the effect of BMD on memory CD3+ T-lymphocytes, tissue fat on CD3+CD40L+ T-lymphocytes, waist size on naive and memory CD3+ T-lymphocytes, memory CD8+ T-lymphocytes and RANK on plasmacytoid DCs.

## 4. Discussion

Several reports have shown the imune system changes in postmenopausal women. The loss of ovarian steroids is associated with menopause and could affect the age related decline in immune function, known as immunosenescence. Immunosenescence is associated with the occurrence of different diseases (e.g., obesity, osteoporosis, infection, malignancy). Endocrinosenescence occurs simultaneously with immunosenescence, and both are associated with profound changes in innate and adaptive immune responses [16,17]. We have observed changes in levels of T- and B-lymphocytes, cytotoxic CD8+ T-lymphocytes, naive and memory T-lymphocytes, plasmacytoid DCs, and CD3+CD40L+ and CD3+CD28+ activated T-lymphocytes. Our finding of diminished frequency of naive T-lymphocytes and increased level of memory T-lymphocytes in postmenopausal women are consistent with other authors [1,12,18]. Decreased number in circulating T and B cells in postmenopausal women may be related to immune senescence. There are variable results in the previous reports demonstrating the occurence of T cell subsets alterations in postmenopausal women (e.g., considerable decreased in CD4+CD8+ ratio, moderate non-significant differences in CD markers) [16,19]. Our finding of the significant reduction in the number of cytotoxic CD8+ T-lymphocytes in postmenopausal women group compared to fertile women is consistent with Engelmann et al. [12]. This decline in number of CD8+ T-lymphocytes can be explained by ageing and the development of autoimmune or infectious diseases, and may reflect T cell exhaustion during chronic low-grade inflammation and an increase of apoptosis.

CD40L is a co-stimulation molecule that belongs to the tumour necrosis factor (TNF) family. CD40L−CD40 interaction is important for T cell effector function and optimal antibody responses by B cells and is involved in bone metabolism [20]. T cells can regulate osteoblastic cells via cell surface receptors such as CD40L [21]. Both T cells and B cells cooperate for maintenance of peak bone mass via production of osteoprotegerin by B cells, and augmentation by T cells. Furthermore, researchers found that bone marrow osteoprotegerin production is stimulated through CD40L-CD40 interactions, and associated to osteoporosis prevention [22,23]. Estrogen deficiency increases the number of activated CD40L-expressing T cells that promote the expression of M-CSF and RANKL by stromal cells and downregulates the production of osteoprotegerin [24]. Therefore, cross-talk between T cells and stromal cells, mediated by CD40L, plays a pivotal role in the dysregulation of osteoblastogenesis and osteoclastogenesis [25]. We observed the reduced levels of CD3+CD40L+ and CD3+CD28+ T-lymphocytes in postmenopausal women compared to fertile women. These results would support the hypothesis of age related immune impairment [26,27]. The decline of the immune system appears to be an intractable consequence of aging, leading to increased susceptibility to infections, reduced effectiveness of vaccination and higher incidences of many diseases including osteoporosis and cancer in the elderly. These outcomes can be attributed, at least in part, to a phenomenon known as T cell replicative senescence, a terminal state characterized by dysregulated immune function, loss of the CD28 costimulatory molecule, shortened telomeres and elevated production of pro-inflammatory cytokines. Therefore, downregulation of CD28 would fundamentally alter normal T cell function, survival, and proliferation [28].

T cells play a role in the pathogenesis of postmenopausal bone loss and indicate relationship between skeletal regulation by estrogen and the immune system [29]. RANKL and its receptor RANK are key regulators of bone remodelling, T cells-DCs communications, and lymph node formation [30]. T cells play an unexpected role in the regulation of bone resorption and bone formation through a variety of mechanisms. There is evidence that human estrogen deficiency might expands to postmenopausal osteoporosis through modifying RANK/RANKL/osteoprotegerin pathway. The emerging role in postmenopausal bone loss has T cell products (e.g., TNF-α, IL-1) [31]. Hematopoietic cells from various developmental stages, including RANK (CD265)+ monocytes, and CD14+ monocytes, possess the ability to undergo osteoclastogenesis. Levels of RANK expression on monocytes significantly correlated with level of osteoclastogenesis in healthy volunteers. The RANKL-RANK interaction is very important as a regulator of the connections between T cells and DCs, and RANK is essential for osteoclastogenesis [32,33,34]. Obesity is traditionally viewed to be beneficial to bone health, despite being a risk factor for many other chronic health disorders. Obese BMI as a protective factor against osteoporosis may be caused by the interaction between fat and bone tissue such as the effect of fat mass on the secretion of bone active hormones from the pancreatic beta-cells (e.g., insulin, amylin) and the secretion of the bone active hormones from adipocytes (e.g., leptin). Now, obesity is considered a risk factor for osteoporosis because obesity promotes inflammation. The increased circulating and tissue proinflammatory cytokines in obesity may promote osteoclast activity and bone resorption through modifying the RANKL/RANK/ osteoprotegerin pathway [35,36]. The underline pathophysiological relationship between obesity and bone is complex and continues to be an active research area. The cross-talk between fat and bone likely constitutes a homoeostatic feedback system in which adipokines and molecules secreted by osteoblasts and osteoclasts represent the link of an active bone-adipose axis. Additionally, in obese post-menopausal women, increased estrogen synthesis by adipose tissue has been suggested as one of the potential mechanisms for the protective effect of fat mass on bone. Zhao et al. [34], for the first time, have shown evidence of linkage and association of the RANK gene with obesity in a large sample size. Their results indicate that genetic variation in this gene may reduce/increase the risk of obesity and osteoporosis in the same direction. Our results showed the influence of obesity on the RANK receptor. We have observed diminished expression of RANK on monocyte population from postmenopausal obese women in comparison to postmenopausal control and negative correlation between waist size and RANK amounts on plasmacytoid DCs. Because the inflammatory osteoclasts can derive from peripheral blood monocytes, we hypothesize that RANKL-induced monocyte migrate from peripheral blood and may contribute to bone loss. The reduced RANK expression may be related also to the induction of ectodomain shedding as a feedback inhibition by inflammatory factors [37,38].

BMD influences the risk of osteoporosis later in life, and the naturally occurring Treg cells not only balance immune activation, but also extend their role in bone homeostasis, thus representing a central cellular mechanism for maintaining bone mass. Treg cells play a crucial role in maintaining peripheral tolerance and decrease the levels of proinflammatory effector T cells [39,40,41]. Treg cells play a critical role in the regulation of osteoclastogenesis and bone resorption. Currently, there is no consensus regarding the mechanism of Treg cells in inhibiting immune responses, they act through the direct killing of cytotoxic cells through cell-to-cell contact, the inhibition of cytokine production by cytotoxic cells, and the direct secretion of immunomodulatory cytokines [42,43,44]. In the present study we observed an enhanced level of CD4+CD25+CD127− Treg cells in women with osteoporosis. Treg cells increased levels may act as a defense against increased inflammation during osteopenia and osteoporosis and may be a pre-indication for chronic illness. The relative increase in circulating Treg cells might play a role in lymphocyte anergy and represent a marker of declining proliferative capacity. In human, these cells are supposed to play a role not only in cancer, autoimmunity, or allergy, but also in infectious diseases [45]. Some authors described an increase in Treg cells during ageing, which however are quite dysfunctional. Suppressive activity of Treg cells declines with age probably of age-dependent thymic atrophy or the senescent peripheral environment [46].

Postmenopausal hormonal changes may modify the function of innate immune cells such as DCs. Plasmacytoid DCs have a pivotal role in various diseases (e.g., SLE, chronic hepatitis C). Estrogens could exert profound modulatory effect through their action on the innate function of plasmacytoid DCs, and they modulate Toll-like receptor (TLR) signaling and have potent regulatory effect in the production of type IIFNs [19,47,48]. Our current study confirms that hormonal changes in postmenopausal women may act on the function of immune cells such as plasmacytoid DCs. Estrogens may regulate key signaling molecules of the TLR pathway, or components implicated in their intracellular trafficking or proteolytic cleavage [49]. The loss of circulatory plasmacytoid DCs can be associated with ageing, response to infections, and accumulation of specific DCs in adipose tissue in human obesity [50]. We demonstrated here the lower level of plasmacytoid DCs in women with osteoporosis and CD3+CD28+ T-lymphocytes in women with osteopenia. The downregulation of DCs may be caused by the sequestration of cell population into inflamed tissue and downregulation of surface markers following activation. The loss of costimulatory molecules CD28 on T-lymphocytes may be related to chronic inflammation and normal aging process [51,52].

Our data suggested that women age, BMI, BMD, waist size and tissue fat may alter cell surface receptor expression and cell population in women (e.g., naive and memory T-lymphocytes, CD3+CD28+ T-lymphocytes, DCs). Waist size seemed to be positively associated with the level of CD4+ T-lymphocytes, which have a critical role in visceral adipose tissue inflammation and increase with age. The CD28 costimulatory molecule is required for lymphocyte activation and development of functional Treg cell compartment. The CD28 deficiency decreased a pathogenic T cells and Treg cell content within adipose tissue [53,54].

Multivariate linear regression models showed that besides the effect of postmenopausal changes in cell surface receptor expression, there is an association between women age and decline of RANK on myeloid DCs. The system of these changes is linked to the pathogenesis of bone volume reduction and postmenopausal osteoporosis. The estrogen deficiency in postmenopausal women facilitates bone loss. A common feature of osteoporosis is a deficit in osteoblastogenesis and osteoblastic activity. This deficit is known to be caused by several factors at the cellular level, including a reduced number of stromal precursors in the bone marrow, a decreased osteoblastogenesis, shortened osteoblast life span, increased adipogenesis, increased osteoblast/osteocyte apoptosis and hormonal alteration [55,56]. We have shown that the expression of CD40L on T-lymphocytes is related to women age and waist size. CD40L is a potential modulator of adipose tissue inflammation, and reveal potent metabolic function aside from its known pivotal proinflammatory role. This molecule plays a crucial role in the development of obesity-induced inflammation and metabolic complications [57,58].

## 5. Conclusions

This paper summarizes differences in cell populations, such as cytotoxic T-lymphocytes, naive T-lymphocytes, and plasmacytoid DCs, between fertile and postmenopausal women. We have described postmenopausal changes in some cell surface marker expression (e.g., CD40L, CD28, CD265). Obesity affected markedly the cell surface expression of RANK in postmenopausal women. Osteoporosis is linked to reduced percentage of plasmacytoid DCs, but elevated natural Treg cells. Our findings indicate that postmenopausal life period might be associated with an aberrant cellular immunity, and we show that co-factors such as women age, BMI, BMD, waist size and tissue fat may influence immunophenotyping data.

## Figures and Tables

**Table 1:** Total cell surface expression and cell populations in blood samples from women.

Cluster of Differentiation (CD) Markers	Mean (%)	Median (%)	Percentiles 25	Percentiles 75
CD3+ T-lymphocytes	71.99	72.5	67.6	76.95
CD4+ T-lymphocytes	45.63	45	39.9	51.2
CD8+ T-lymphocytes	20.26	20	20	23.93
CD19+ B-lymphocytes	9.49	9.1	7.28	11.63
CD19+HLADR+HLADR+ B-lymphocytes	9.17	8.75	6.98	11.13
CD3−CD56+ NK cells	10.86	9.65	6.5	14.33
CD3+CD56+ NKT cells	5.04	3.2	1.85	6.4
CD3+CD28+ Activated T-lymphocytes	58.87	59.8	51.5	66.23
CD3+CD40L Activated T-lymphocytes	8.34	7.95	6.1	10
CD4+CD127+CD25− Memory effector T cells	86.04	87.9	83.95	90.63
CD4+CD127−CD25+ Natural regulatory T-cells	3.06	2.85	1.78	4.13
CD3+CD45RO−CD45RA+ Naive CD3+ T-lymphocytes	35.56	35.2	28.38	42.6
CD3+CD45RO+CD45RA− Memory CD3+ T-lymphocytes	45.92	43.45	36.98	49
CD3+CD8+CD45RO−CD45RA+ Naive CD8+ T-lymphocytes	38.51	39.5	30.68	47.33
CD3+CD8+CD45RO+CD45RA− Memory CD8+ T-lymphocytes	31.45	30.6	24	37.28
CD19−HLADR+CD123+ Plasmacytoid dendritic cells	0.63	0.6	0.4	0.8
CD19−HLADR+CD11c+ Myeloid dendritic cells	0.52	0.5	0.3	0.7
CD19−HLADR+CD11c−CD123+ Plasmacytoid Dendritic cells	14.72	13.6	8.98	18.85
CD19−HLADR+CD11c+CD123− Myeloid Dendritic cells	9.41	7.75	5.48	11.2
CD265+ (RANK) Monocyte population	4.84	4.7	3.68	5.9
CD265+CD11c+ Monocyte population	1.73	1.6	1.1	2.13
CD265+CD19−HLADR+CD11c+CD123− RANK on Myeloid Dendritic cells	11.18	10.5	5.7	15.4
CD265+CD19−HLADR+CD11c−CD123+ RANK on Plasmacytoid Dendritic cells	15.05	13.3	13.3	21
CD3+CD254+ RANKL on T-lymphocytes	3.53	3.3	2.4	4.6

**Table 2:** Comparison of cell surface receptors and cell populations in whole blood samples from fertile and postmenopausal women.

CD Markers	Fertile Women		Postmenopausal Women		Significance
	1. FC	2. FO	3. PMC	4. PMO	
CD3+ T-lymphocytes		•		•	*p* < 0.05
CD19+ B-lymphocytes	•		•		*p* < 0.05
CD3+CD45RA+CD45RO− Naive CD3+ T-lymphocytes		•		•	*p* < 0.05
	•		•		*p* < 0.05
CD19-HLADR+CD123+ Plasmacytoid Dendritic cells			•	•	** *p* < 0.01
		•		•	*p* < 0.05
CD265+ (RANK) Monocyte population			•	•	** *p* < 0.05
		•		•	*p* < 0.05
CD265+CD11c+ Monocyte population			•	•	** *p* < 0.01
		•		•	*p* < 0.01
CD3+CD40L Activated T-lymphocytes		•		•	*p* < 0.05
CD3+CD28+ Activated T-lymphocytes		•		•	*p* < 0.05

Women were divided into four groups: 1. FC = fertile control, 2. FO = fertile obese women, 3. PMC = postmenopausal control, and 4. PMO = postmenopausal obese women. Statistical significance *p* < 0.05, *p* < 0.01; ** = influence of BMI factor; CD = cluster of differentiation; BMI = body mass index. • the comparison of the groups with a significant difference. The lower percentages of cells were in postmenopausal women (PMO and PMC).

**Table 3:** CD markers correlation with waist size, BMD, tissue fat and BMI in the total study cohort.

CD Markers	BMI	Tissue Fat	Waist Size	BMD	Age
CD4+ T-lymphocytes			r = 0.149 *p* < 0.05		
CD19−HLADR+CD123+ Plasmacytoid Dendritic cells	r = −0.178 *p* < 0.05	r = −0.195 *p* < 0.01	r = −0.159 *p* < 0.05		
CD3+CD28+ Activated T-lymphocytes		r = −0.145 *p* < 0.05		r = −0.153 *p* < 0.05	r = −0.157 *p* < 0.05
CD265+CD19−HLADR+CD11c−CD123+ RANK on Lymphoid Dendritic cells			r = −0.196 *p* < 0.05		
CD4+CD127+CD25− Memory effector cells				r = −0.172 *p* < 0.05	
CD3+CD45RO+CD45RA− Memory CD3+ T-lymphocytes				r = 0.207 *p* < 0.01	
CD3+CD45RO−CD45RA+ Naive CD3+ T-lymphocytes					r = −0.229 *p* < 0.01
CD19−HLADR+CD11c+ Myeloid Dendritic cells					r = −0.152 *p* < 0.05

BMI = body mass index; BMD = bone mineral density; CD = cluster of differentiation; r = correlation coefficient; statistical significance *p* < 0.05, *p* < 0.01.

**Table 4:** Coefficients and standard errors in models predicting cell surface markers in samples of blood from fertile and postmenopausal women.

Covariantes	Models Predicting Percentage of:								
	CD4+			CD3+			CD19+		
	B	SE	Sig.	B	SE	Sig.	B	SE	Sig.
Women									
fertile vs.	4.091	2.968	0.170	4.662	2.621	0.077	−1.606	1.088	0.142
postmenopausal									
BMD	−0.029	1.259	0.982	−0.337	1.112	0.762	0.244	0.461	0.597
BMI	0.054	0.266	0.838	−0.069	0.235	0.771	−0.088	0.097	0.369
Tissue fat	−0.041	0.157	0.796	0.051	0.138	0.714	0.032	0.057	0.573
Waist size	0.106	0.115	0.358	0.030	0.101	0.770	0.034	0.042	0.418
Women age	−0.088	0.114	0.439	−0.227	0.101	*p* < 0.050	0.040	0.042	0.343
	**CD3+CD45RO+CD45RA−**			**CD3+CD45RA+CD45RO−**			**CD3+CD8+CD45RO+CD45RA−**		
	**B**	**SE**	**Sig.**	**B**	**SE**	**Sig.**	**B**	**SE**	**Sig.**
Women									
fertile vs.	11.135	15.253	0.466	−6.919	3.938	0.081	4.196	3.88	0.281
postmenopausal									
BMD	16.457	6.617	*p* < 0.050	2.040	1.708	0.234	−0.175	1.685	0.917
BMI	0.699	1.344	0.604	−0.395	0.347	0.256	0.169	0.342	0.622
Tissue fat	1.059	0.799	0.187	−0.103	0.206	0.617	0.271	0.203	0.185
Waist size	−1.153	0.579	*p* < 0.050	0.322	0.150	*p* < 0.050	−0.298	0.148	*p* < 0.050
Women age	−0.539	0.590	0.362	−0.039	0.152	0.799	−0.084	0.150	0.576
	**CD3−CD56+**			**CD265+CD19−HLADR+CD11c+CD123-**			**CD3+CD40L+**		
	**B**	**SE**	**Sig.**	**B**	**SE**	**Sig.**	**B**	**SE**	**Sig.**
Women									
fertile vs.	1.844	2.080	0.377	5.698	2.685	*p* < 0.050	−3.323	1.574	*p* < 0.050
postmenopausal									
BMD	−0.367	0.900	0.684	−0.377	1.138	0.741	0.123	0.682	0.857
BMI	0.21	0.183	0.249	−0.101	0.243	0.678	0.124	0.141	0.381
Tissue fat	−0.068	0.109	0.533	0.234	0.143	0.105	−0.179	0.083	*p* < 0.050
Waist size	−0.050	0.079	0.524	−0.069	0.108	0.521	0.037	0.060	0.539
Women age	−0.015	0.08	0.851	−0.242	0.104	*p* < 0.050	0.131	0.061	*p* < 0.050
	**CD3+CD56+**			**CD265+CD19−HLADR+CD11c−CD123+**			**CD3+CD8+**		
	**B**	**SE**	**Sig.**	**B**	**SE**	**Sig.**	**B**	**SE**	**Sig.**
Women									
fertile vs.	−1.594	2.061	0.440	−2.662	3.842	0.490	0.482	2.360	0.838
postmenopausal									
BMD	−0.073	0.874	0.933	1.900	1.622	0.243	−0.004	1.001	0.997
BMI	0.236	0.185	0.205	0.539	0.347	0.122	−0.165	0.211	0.436
Tissue fat	−0.108	0.109	0.322	0.059	0.205	0.773	0.030	0.125	0.809
Waist size	−0.068	0.080	0.396	−0.411	0.154	*p* < 0.010	0.000	0.091	0.999
Women age	−0.099	0.079	0.213	0.060	0.149	0.689	−0.065	0.091	0.474

Percentage of: CD265+CD19-HLADR+CD11c+CD123− cells = 5.698 women fertile and postmenopausal −0.242 women age; CD3+CD40L+ cells = −3.323 women fertile and postmenopausal −0.179 tissue fat 0.131 women age (*p* < 0.05 for all independent variables included in the equation). B = coefficients. SE = standard error. BMI = body mass index. BMD = bone mineral density. Sig. = statistical significance

## Abbreviations

BMI	Body Mass Index
BMD	Bone Mineral Density
CD	Cluster of Differentiation
DCs	Dendritic Cells
NK cells	Natural Killer cells
NKT cells	Natural Killer T cells
Treg cells	Regulatory T cells
RANK	Receptor Activator of Nuclear Factor κ-B
RANKL	Receptor Activator of Nuclear Factor κ-B Ligand
GFR	Glomerular Filtration Rate
TLR	Toll-Like Receptor
